# Regulation of alternative splicing by retrograde and light signals converges to control chloroplast proteins

**DOI:** 10.3389/fpls.2023.1097127

**Published:** 2023-02-10

**Authors:** Guiomar Martín

**Affiliations:** Department of Biology, Healthcare and the Environment, Faculty of Pharmacy and Food Sciences, University of Barcelona, Barcelona, Spain

**Keywords:** alternative splicing, retrograde signaling, light signaling, chloroplasts, nonsense-mediated decay RNA pathway, molecular convergence, *Arabidopsis thaliana*

## Abstract

Retrograde signals sent by chloroplasts control transcription in the nucleus. These signals antagonistically converge with light signals to coordinate the expression of genes involved in chloroplast functioning and seedling development. Although significant advances have been made in understanding the molecular interplay between light and retrograde signals at the transcriptional level, little is known about their interconnection at the post-transcriptional level. By using different publicly available datasets, this study addresses the influence of retrograde signaling on alternative splicing and defines the molecular and biological functions of this regulation. These analyses revealed that alternative splicing mimics transcriptional responses triggered by retrograde signals at different levels. First, both molecular processes similarly depend on the chloroplast-localized pentatricopeptide-repeat protein GUN1 to modulate the nuclear transcriptome. Secondly, as described for transcriptional regulation, alternative splicing coupled with the nonsense-mediated decay pathway effectively downregulates expression of chloroplast proteins in response to retrograde signals. Finally, light signals were found to antagonistically control retrograde signaling-regulated splicing isoforms, which consequently generates opposite splicing outcomes that likely contribute to the opposite roles these signals play in controlling chloroplast functioning and seedling development.

## 1 Introduction

During all their life cycle, plants adjust gene expression to produce the protein set that fulfills the demands of each environment and developmental stage. Regulation of transcription is key to control gene expression in response to external and internal cues. These cues modulate the capacity of transcription factors to bind specific cis-regulatory DNA regions and thus, adjust the expression levels of messenger RNA precursors (pre-mRNAs). In addition, gene expression is regulated by post-transcriptional mechanisms that control the transcript processing, nuclear export, translation and/or stability. Processing of pre-mRNAs includes the removal of introns and the subsequent ligation of the flanking exons to produce mature mRNAs (mRNAs). This molecular process is called splicing and is carried out by the spliceosome, a large ribonucleoprotein complex that recognizes the splice sites at the intron-exon boundaries (Matera and Wang, 2014). However, not every splice site is recognized every time a gene is transcribed, leading to alternative splicing (AS). In conjunction with the spliceosomal proteins, associated RNA-binding proteins also regulate splicing by binding to enhancer or silencer cis-regulatory elements in the pre-mRNA (Martinez-Contreras et al., 2007; Long and Caceres, 2008; Wang and Burge, 2008). Complementarily, different genomic features influence the spliceosome’s capacity of recognizing the splice sites and, subsequently, the definition of introns and exons. These include the splice site’s strength (i.e., how near to consensus is their sequence), the exon and intron size, or their GC content (Sterner et al., 1996; Amit et al., 2012; de Conti et al., 2013). Four major types of AS events can occur on a particular transcript: retention of introns (IR), skipping of exons (ES) and alternative selection of 5´ and 3´ splice sites (ALT5 and ALT3), all of them giving rise to alternative mRNA isoforms. Given that 5´ and 3´ untranslated regions (UTRs) play important roles in the post-transcriptional control of gene expression (Srivastava et al., 2018), AS events located in these regions will potentially affect this regulation. Moreover, depending on the size and nucleotide composition of the alternative sequences, AS events located in the gene coding region (CDS) can either preserve or not the reading frame, which will consequently produce alternative isoforms or truncated proteins. In many cases, reading frame shifts generated by AS, as well as the inclusion of intronic sequences, introduce premature stop codons (PTCs), which in turn trigger mRNA degradation through the nonsense-mediated decay RNA pathway (NMD; Chang et al., 2007; Isken and Maquat, 2007). Coupling of AS to NMD allows eukaryotes to modulate gene expression by balancing the ratio between productive and unproductive mRNA isoforms (Kalyna et al., 2012; Drechsel et al., 2013). Different studies have revealed the importance of this coupling for the proper implementation of particular developmental processes and environmental responses (Lewis et al., 2003; Wong et al., 2013; Hartmann et al., 2016).

In recent years, multiple genome-wide studies have profiled AS landscapes in *Arabidopsis thaliana*, especially of plants subjected to different environmental signals, mostly associated to abiotic stress (Mastrangelo et al., 2012; Laloum et al., 2018). These plant studies have consistently shown that IR and ALT3 are the most common types of AS events, and also, the higher contribution of AS in modulating gene expression levels compared to expanding the proteome (Chaudhary et al., 2019; Martín et al., 2021). Despite the wide variety of environmental signals studied, it is still not known how plastid-to-nucleus retrograde signals influence nuclear AS genome-wide. These signals, together with anterograde signals from nucleus to chloroplasts, are needed to coordinate the expression of chloroplast proteins, encoded by both nuclear and chloroplast genomes (Griffin and Toledo-Ortiz, 2022). Retrograde signals are strongly dependent on the chloroplast-localized pentatricopeptide-repeat protein GUN1 (Susek et al., 1993; Koussevitzky et al., 2007), and are key during chloroplast biogenesis (biogenic control) and to adjust photosynthesis rates to the environment (operational control) (Barajas-López et al., 2013). As retrograde signaling (RS) robustly downregulates nuclear transcription of chloroplast genes (commonly named photosynthesis-associated nuclear genes*; PhANGs*; Allen et al., 2003), this molecular readout has been extensively used to establish the nature of retrograde signals and their signaling components. Despite the substantial knowledge on RS regulation at the transcriptional level, which has firmly demonstrated a molecular convergence with the light signaling pathway (Ruckle et al., 2007; Ruckle et al., 2012; Martín et al., 2016; Xu et al., 2016), very little is known about its effect on AS. In fact, evidences of RS-mediated AS in Arabidopsis have only been reported for three genes (Petrillo et al., 2014).

Using publicly available RNA sequencing data, this study addresses the impact of norflurazon (NF), a plant herbicide that activates retrograde signaling (Oelmüller et al., 1986), on AS. This strategy enabled a comprehensive characterization of the genes differentially spliced in response to RS, as well as of the molecular and biological functions of this regulation. Similarly to the RS-mediated nuclear regulation of transcription, AS regulation was revealed to be dependent on GUN1. Moreover, these results indicate that retrograde signals disturb the spliceosome’s capacity to properly splice introns and exons associated with specific genomic features. In most cases the RS-induced alternative mRNA isoform is committed to degradation through the NMD surveillance pathway, thus downregulating the total transcript levels. Interestingly, genes that undergo this type of regulation are enriched for genes encoding chloroplast proteins. Therefore, in response to RS, AS acts along with transcription to effectively downregulate the expression of this type of proteins. Finally, these results indicate that light signals also control splicing of the RS-regulated AS events, which implies that, as established for the transcriptional control of gene expression, AS regulation also converges downstream of light and retrograde signals.

## 2 Material and methods

### 2.1 Definition of differentially expressed genes in response to norflurazon

Quantification of Arabidopsis total mRNA levels from public sequencing data (GSE110125; Xiaobo et al., 2019) was performed using *vast-tools* v.5.1. For each Arabidopsis transcript, this tool provides the corrected-for-mappability RPKMs (cRPKMs), which represents the number of mapped reads per million mapped reads divided by the number of uniquely mappable positions of the transcript (Labbé et al., 2012). To compare the expression of wild-type (WT) seedlings treated or not with norflurazon, *vast-tools_compare_expr* command was employed with the option -norm, which allows a quantile normalization of cRPKMs between samples. Moreover, genes that had read counts < 50 and were not expressed at cRPKM > 5 across all replicates of at least one of the two samples compared were filtered out. Finally, those genes with a fold change of at least 2 between each of the individual replicates from each sample analyzed were defined as differentially expressed genes (NF-regulated genes; **Supplemental Table 1**). See https://github.com/vastgroup/vast-tools for details.

### 2.2 Definition of differentially spliced events in response to norflurazon, lincomycin and light

*Vast-tools* (Tapial et al., 2017) was employed to quantify alternative splicing from each individual sample of three public RNA-seq experiments: GSE110125 (Xiaobo et al., 2019), GSE130337 (Xu et al., 2020) and GSE164122 (Martín and Duque, 2021), which respectively address responses to norflurazon, lincomycin and light in *Arabidopsis thaliana* seedlings. This tool maps RNA-seq data to the araTha10 library, based on Ensembl Plants v31 and composed by an extended annotation with all exon-exon and exon-intron junction sequences found in the *Arabidopsis thaliana* genome using a large compendium of RNA-seq datasets (see Martín et al., 2021 for details). The mapping and experimental details of the samples used in the current study are summarized in **Supplemental Table 2**. Then, *vast-tools* quantifies ES, IR, ALT5 and ALT3 of each sample analyzed, and provides the percent of inclusion (PSI; Percent of Spliced In) of each putative alternative sequence using only exon-exon (or exon-intron for IR) junction reads (Braunschweig et al., 2014; Tapial et al., 2017). In addition, *vast-tools* associates a quality score to each AS event based on the read coverage that sustains the PSI quantification. To define differentially spliced events in response to norflurazon, lincomycin or light, I used the command *vast-tools compare* adding specific filters. First, VLOW events were discarded, which represent the lowest range of read coverage (for further details see https://github.com/vastgroup/vast-tools). To improve the read coverage that supports each splicing junction and thus decrease the number of AS events discarded by this filter, replicates of each sample were pulled together (using the *vast-tools merge* function). The consistency of these merged samples was confirmed (**Supplemental Figure 1**). Moreover, to evaluate up and down sequence inclusion of all ALT events, not only of the most external splice sites, the analysis was conducted with the –legacy_ALT *vast-tools compare* option. Additionally, the –p_IR and –min_ALT_use 25 options were also applied. The first eliminates those IR events with a significant imbalance between the two exon-intron junctions (*P* < 0.05; binomial test; see Braunschweig et al., 2014 for details). –min_ALT_use 25 ensures that ALT3 and ALT5 events are located in exons with a sufficient inclusion level, in particular, implies that the host exon has a minimum PSI of 25 in each compared sample. Then, those splicing events with a |ΔPSI| > 15 between the pair of samples being compared were selected as differentially spliced events (NF-, lincomycin- or light-regulated AS events; respectively found in **Supplemental Tables 3**-**5**). Furthermore, genes exhibiting differential AS were considered differentially spliced genes (AS genes). Then, the pertinent control sets were generated to conduct molecular and functional analyses of the AS events and AS genes. First, non-regulated AS events were those that passed the coverage criteria and were not differentially spliced (|ΔPSI| < 15). Genes for which all their AS events belong to the group of non-regulated events constituted the group of non-regulated AS genes; by definition, these genes are all multiexonic.

### 2.3 Inclusion values of norflurazon-regulated AS events in multiple experimental conditions

**Figure 1:**
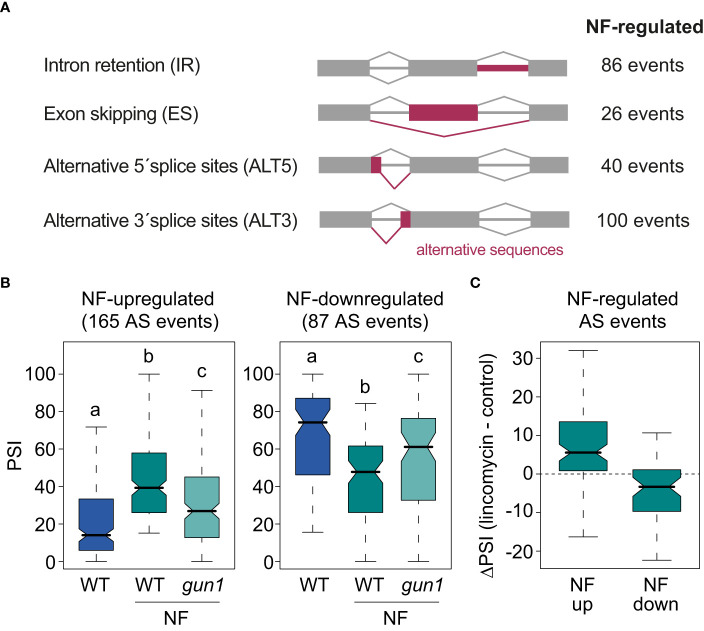
AS events regulated by retrograde signals. **(A)** Schematic representation of the major types of AS events and the number of AS events of each class regulated by norflurazon (NF). **(B)** Percent of inclusion values (PSI) of the alternative sequences of the 165 NF-upregulated (left) and the 87 NF-downregulated (right) AS events in wild-type (WT) and *gun1* samples treated or not with NF. Letters denote the statistically significant differences between medians (Kruskal-Wallis; *P* < 0.05). **(C)** Boxplot representation of the ΔPSI between WT samples treated or not with lincomycin of AS events up- or downregulated by NF.

Three different publicly available RNA-seq experiments were analyzed with *vast-tools* to quantify the PSI values of NF-regulated AS events in three different conditions. First, GSE130337 (Xu et al., 2020), to address the molecular response to lincomycin in Arabidopsis seedlings (**Figure 1C**). GSE164122 (Martín and Duque, 2021) was used to study light responses of dark-grown seedlings (**Figure 6C**), and finally, GSE41432 (Drechsel et al., 2013), to show the PSI values in WT, *upf1*, *upf3* and *upf1upf3* seedlings (**Figure 4C**). The mapping and experimental details of the samples used in the current study are summarized in **Supplemental Table 2**.

### 2.4 Percentage of response to norflurazon in the *gun1* mutant

For each AS event and gene, the ΔPSI and the logarithmic to base 2-fold change were calculated comparing either WT or *gun1* norflurazon-treated seedlings with untreated WT seedlings. This value represents the magnitude of change in expression or splicing in response to retrograde signals. To compare WT and *gun1* values, the WT value was set to 100, to then calculate the percentage of response in the *gun1* based on this equivalence. This is, *gun1* value multiplied by 100 and then, divided by the WT value. The resulting percentage indicates how much of the molecular response to norflurazon occurring in the WT is kept in the *gun1* mutant.

### 2.5 Molecular analysis of differentially spliced sequences

Alternatively spliced sequences were classified based on their localization within the gene structure: 5´-UTR, CDS and 3´-UTR. In addition, the predicted impact on the canonical open reading frame was mainly categorized in function of whether or not they preserve the reading frame (i.e., they have length multiple of three nucleotides) and contain in-frame stop codons predicted to trigger NMD (stop codons considered premature because they are located at least 50 nucleotides upstream of an exon-exon junction; see Martín et al., 2021 for details). These alternative possibilities generate three different scenarios, AS events that produce alternative protein isoforms, or unproductive transcripts when the alternative sequence is included or excluded. This information was retrieved from the download section of PastDB (Legacy version; http://pastdb.crg.eu/wiki/Downloads; Plant alternative splicing and transcription Data Base). Moreover, comparison of exon and intron features associated with splicing regulation was performed with Matt v1.3.0 (Gohr and Irimia, 2019). For each compared group of exons and introns events: upregulated, downregulated and non-regulated, this tool obtained and compared the exon and intron length, the GC content and the splice site strength (see Martín et al., 2021 for details regarding calculations of splicing site strength).

### 2.6 Functional analysis of genes differentially spliced

NF-regulated AS genes were functionally categorized according to their subcellular localization and gene ontology (GO) annotation. A single subcellular localization was assigned to each locus based on the annotation of the Arabidopsis genome (Araport 11) and percentages of each type were plotted discarding those genes with unknown subcellular localization. To identify enriched GO biological processes, molecular functions and cellular components, analyses were performed using the functional annotation classification system DAVID (Huang et al., 2007). The same strategy was used to define GO category enrichment of lincomycin-regulated AS genes.

## 3 Results

### 3.1 AS regulation in response to retrograde signals is dependent on GUN1

To define how retrograde signals control splicing, the AS profiles of WT seedlings grown in the presence or absence of norflurazon were compared (GSE110125; Xiaobo et al., 2019). This herbicide inhibits carotenoid biosynthesis and causes accumulation of Mg-protoporphyrin IX, which acts as a retrograde signaling (Oelmüller et al., 1986; Strand et al., 2003). First, *vast-tools* (Tapial et al., 2017) was used to quantify steady-state mRNA levels in each sample (hereafter gene expression; GE), and to determine changes in expression levels of representative *PhANGs* (Allen et al., 2003). As expected, all *PhANGs* were consistently downregulated in the WT in response to norflurazon (**Supplemental Figure 2**). Moreover, in line with the key role of GUN1 mediating the transcriptional changes induced by RS (Koussevitzky et al., 2007), this downregulation was weaker in the *gun1* mutant (**Supplemental Figure 2**). Additionally, 94.5% of the genes described as NF-regulated in this analysis (**Supplemental Table 1**) were also identified as NF-regulated in Xiaobo et al., 2019 (**Supplemental Figure 3**). These results thus confirm the quality of this GE quantification.

Then, *vast-tools* was used to define alternatively spliced genes. This tool quantifies the sequence inclusion levels for all four major AS types (IR, ES, ALT5, ALT3; **Figure 1A** and Material and Methods for details). For each sample and splicing event in the genome, *vast-tools* provides the percent of alternative sequence inclusion (using the PSI metric), which corresponds to the percentage of expressed transcripts that include the alternative sequence. The comparison between WT seedlings treated or not with norflurazon retrieved 252 differentially spliced AS events (|ΔPSI| > 15; **Figure 1A** and **Supplemental Table 3**) in 205 genes. As commonly observed in plants, the majority of these events were IR and ALT3 events (Martín et al., 2021). For 165/252 AS events, the alternative sequence was more included in the mRNAs in the presence of norflurazon (NF-upregulated), while for the remaining 87 AS events it was less included (NF-downregulated) (**Figure 1B**). Interestingly, AS changes were also milder in the *gun1* mutant (**Figure 1B** and **Supplemental Figure 4**). In fact, 66% of the NF-regulated AS events in WT plants were not differentially spliced when comparing untreated WT seedlings with NF-treated *gun1* mutants (|ΔPSI| < 15), indicating the implication of this protein in triggering the downstream AS regulation. As observed in **Supplemental Figure 5**, the molecular response to norflurazon in the *gun1* mutant was around half of the response observed in the WT, both for GE and AS. This finding indicates that similarly to the transcriptional control that RS exerts in the nucleus, GUN1 is also key for the nuclear control of AS. Finally, to further confirm that retrograde signals impact splicing of these AS events, their PSI was compared between samples treated or not with lincomycin, an herbicide also known to activate retrograde signals by inhibiting plastid translation (Oelmüller et al., 1986). This analysis showed that inclusion of AS events upregulated or downregulated by the effect of norflurazon was similarly regulated by lincomycin (**Figure 1C**). Therefore, these data demonstrate that retrograde signals control splicing of a subset of transcripts in a GUN1-dependent manner.

### 3.2 RS-regulated AS events share specific genomic features

**Figure 2:**
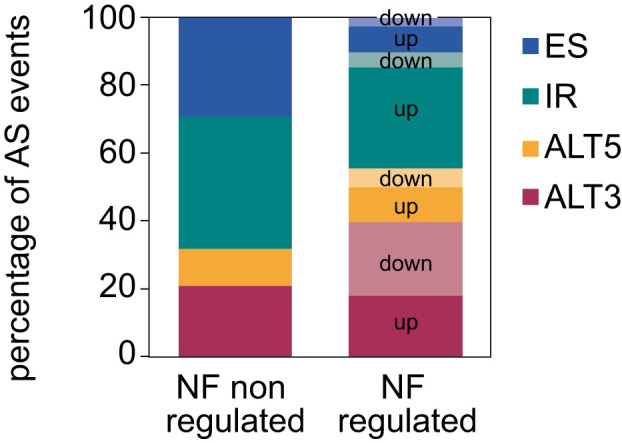
Distribution of the different types of AS events regulated by norflurazon. Number of each type of AS events for which inclusion is differentially up- or downregulated by norflurazon (NF). NF non-regulated AS events represent the proportion of the different types of AS events in the genome (see Material and Methods for details). ALT5, alternative 5´splice site; ALT3, alternative 3´splice site; IR, intron retention; ES, exon skipping.

**Figure 3:**
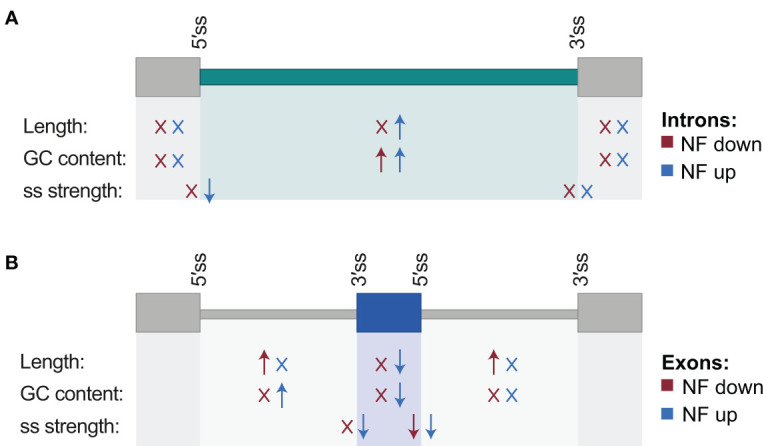
Schematic representation of genomic regulatory features associated with differentially spliced introns and exons. For introns **(A)** and exons **(B)**, arrows summarize which features show statistically significant differences with respect to their control sets of non-regulated introns and exons, and the direction of these differences (higher or lower medians; Mann-Whitney U test; *P* < 0.05). “X” indicates no statistically significant difference. Features include length of the alternatively spliced sequence together with its downstream and upstream exons/introns, GC content, and splicing site strength of their 5´ and 3´ splice sites. Differences are indicated separately for events for which the inclusion of the alternative sequence is down- (red) and up- (blue) regulated by norflurazon (NF). Associated values are shown in **Supplemental Figure 7**.

Next, the proportion of AS events whose inclusion is either up- or downregulated by retrograde signals among each type of AS event was calculated. This uncovered that most of the IR events are upregulated in response to norflurazon, while for the other types of AS events this proportion is similar (**Figure 2**), meaning that RS has a major role in enhancing the inclusion of intronic sequences. Accordingly, this pattern was also characteristic of lincomycin-treated seedlings (**Supplemental Figure 6** and **Supplemental Table 4**). Because genomic features such as the intron and exon length, their GC content or the strength of their splice sites modulate their splicing, characterization and comparison of these regulatory features between NF-regulated introns and exons with those non-regulated was conducted using the Matt software. This study revealed that NF-upregulated introns, those whose splicing fails in the presence of norflurazon (retained), are longer and have weaker 5’ splice sites (**Figure 3A**; **Supplemental Figures 7, 8**), characteristics known to difficult their spliceosomal recognition (de Conti et al., 2013). On the other hand, exons skipped in response to norflurazon (NF-downregulated) tend to be shorter and are surrounded by longer introns (**Figure 3B**; **Supplemental Figures 7, 8**), a pattern also known to hamper their recognition (Hollander et al., 2016). Also, according to their alternative nature, both subsets of NF-regulated exons have weaker splice sites (**Figure 3B**; **Supplemental Figures 7**, **8**). Moreover, the GC content of differentially spliced introns was higher than that of non-regulated introns (**Figure 3A**; **Supplemental Figures 7**, **8**), which implies a reduction of the existing difference between the GC content of introns (lower) and exons (higher), and therefore an additional complication for their recognition (Amit et al., 2012). This reduction is also characteristic of differentially spliced exons, especially of the NF-upregulated subgroup (**Figure 3B**; **Supplemental Figures 7**, **8**). Thus, this analysis detected the genomic particularities shared by the RS-regulated AS events, and revealed that retrograde signals impact the spliceosome’s capacity to properly splice introns and exons imbibed in complex genomic contexts.

### 3.3 RS regulation of AS enhances accumulation of unproductive mRNA isoforms

**Figure 4:**
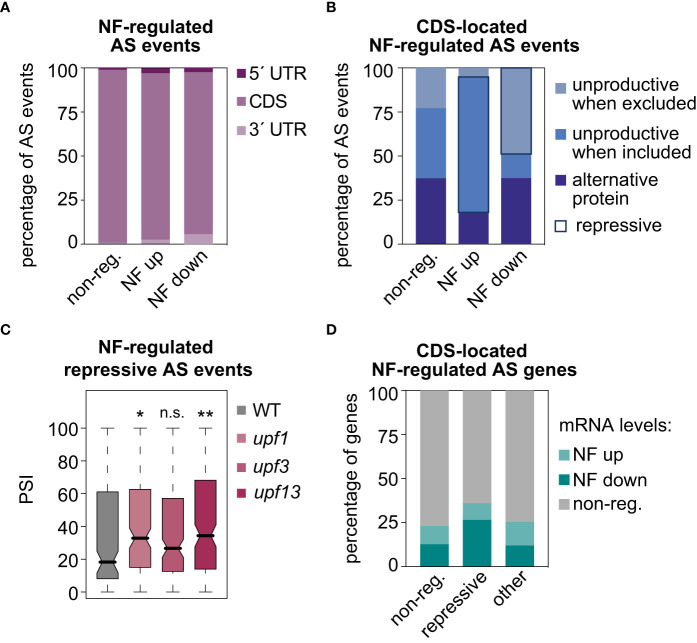
Molecular impact of AS events regulated by retrograde signals. Percentage of AS events located in untranslated (UTRs) or gene coding (CDS) regions **(A)** and percentage of CDS-located AS events that potentially produce unproductive mRNAs or alternative protein isoforms (see Material and Methods for details) **(B)**, in three different groups of AS events: non-regulated (non-reg.), up- or downregulated by norflurazon (NF). Repressive AS events are highlighted. **(C)** Boxplot representation of the percent of inclusion (PSI) values of the NF-regulated repressive AS events in wild-type (WT), *upf1*, *upf3* and *upf1upf3* seedling samples. Asterisks indicate statistically significant differences in respect of the WT (Mann-Whitney U test; *, *P* < 0.05; **, *P* < 0.01; n. s., non-significant). **(D)** Percentage of genes regulated or not by splicing, harboring repressive AS events or other types of CDS-located AS events (other), that belong to our set of differently expressed genes in response to norflurazon (see Material and Methods for details in the gene expression analysis).

Then, the molecular functions of the AS events upregulated or downregulated in response to norflurazon were addressed. As expected given the dominant proportion of the CDS region within the gene structure, the majority of NF-regulated AS events were located in this region (**Figure 4A**). However, despite this predominant presence in the CDS regions, a significant increase in the proportion of AS events located in the UTRs, both in the subsets of NF-upregulated and NF-downregulated AS events, could also be detected (**Figure 4A**; two-sided Fisher’s test; *P* < 0.0012). Focusing on the CDS-located AS events, their impact on the canonical open reading frames (ORFs) was next assessed. In comparison to non-regulated AS events, those events whose alternative sequence is more included in response to norflurazon (NF-upregulated) were enriched for AS events predicted to generate unproductive mRNAs when the alternative sequence is included (**Figure 4B**; i.e., events that introduce a PTC; see Material and Methods for details). Conversely, most of the NF-downregulated AS events generate unproductive mRNAs when the alternative sequence is excluded (**Figure 4B**). Therefore, 67% of the CDS-located NF-regulated AS events have the ultimate effect of enhancing accumulation of unproductive mRNAs when retrograde signals take place (repressive AS events; **Figure 4B**), compared to only 25% predicted to generate alternative protein isoforms, and to a minimal group of AS events (8%) that generate unproductive mRNA isoforms in the absence of norflurazon (**Figure 4B**). Accordingly, among the three categories, repressive AS events also represent the majority of the CDS-located lincomycin-regulated AS events (53%). Because repressive AS events are mainly characterized by carrying NMD-eliciting features, their inclusion levels (PSI) in the Arabidopsis *upf* mutants were quantified (GSE41432; Drechsel et al., 2013). UPF1 and UPF3 genes encode for the two key NMD factors (Hori and Watanabe, 2005; Arciga-Reyes et al., 2006). This analysis globally revealed higher inclusion levels of repressive AS events in these mutants, particularly in the double *upf1upf3* mutant (**Figure 4C**), meaning that their transcript isoforms, accumulated in response to RS, are degraded through the NMD surveillance pathway. In contrast, as expected for AS events predicted to generate alternative proteins, these isoforms lacked NMD regulation (**Supplemental Figure 9**). Therefore, this result implies that RS, through the action of repressive AS events, post-transcriptionally downregulates the mRNA levels of specific transcripts. In agreement, particularly genes harboring repressive AS events are significantly enriched for genes whose mRNA levels are differentially downregulated by norflurazon (**Figure 4D**; **Supplemental Figures 10**, **11**; two-sided Fisher’s test; *P* < 2.5e-5; see *Material and Methods* for details on the GE analysis).

### 3.4 Unproductive mRNA isoforms are functionally linked to chloroplasts

**Figure 5:**
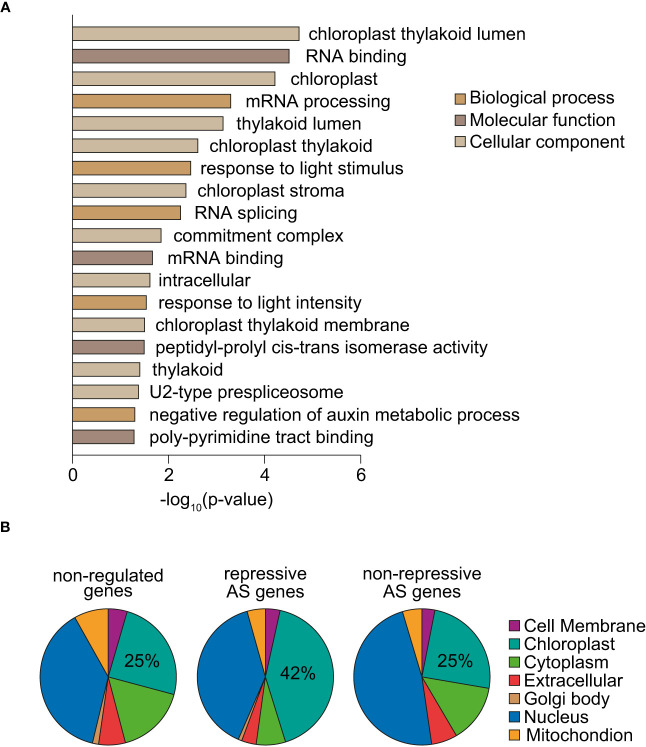
Functional analysis of genes differentially spliced in response to norflurazon. **(A)** Enriched gene ontology categories of the 205 genes defined as differentially spliced in response to norflurazon. DAVID p-value indicates significance (Fisher’s exact test; *P* < 0.05). **(B)** Subcellular localization of genes non-regulated by AS in response to norflurazon (non-regulated) and harboring repressive AS events or other types of RS-regulated AS events (non-repressive). The subcellular classification is based on the Araport 11 predictions available at TAIR (http://www.arabidopsis.org). The percentage of genes encoding for chloroplast-located proteins for each set is indicated.

To gain insight into the biological functions of the 205 genes whose splicing is regulated by norflurazon, a gene ontology (GO) analysis was first conducted. This revealed significant enrichment for chloroplast- and splicing-related GO terms as well as for genes whose expression is light-regulated (**Figure 5A**). A great example of splicing-related genes are those encoding the spliceosome proteins *U2AF65A* and *U2AF65B* (Chen and Moore, 2015), and the splicing regulators *RS2Z32* (Barta et al., 2010) and *GRP8* (Streitner et al., 2012). Additionally, genes from different chloroplast-related categories were found. For example, *PDM2*, *GC1* and *PPL1*, all of them with key roles in chloroplast development and functioning (Maple et al., 2004; Du et al., 2017; Che et al., 2020); *KAC1* and *KAC2*, involved in the movement of chloroplast through actin filaments (Suetsugu et al., 2010); and also, *TIC100*, a component of the TIC chloroplast transport complex recently implicated in RS (Kikuchi et al., 2013; Loudya et al., 2022). Supporting the impact that RS exert on the splicing pattern of this type of genes, these GO terms were also enriched among the subset of genes differentially spliced in response to lincomycin (**Supplemental Figure 12**). Next, classification of NF-regulated AS genes based on the subcellular locations of their encoded proteins revealed overrepresentation of chloroplast-located proteins (**Supplemental Figure 13**; two-sided Fisher’s test; *P* < 0.00074). Strikingly, this enrichment was specific of genes harboring repressive AS events (**Figure 5B**), which represent the majority of RS-regulated AS events (**Figure 4B**). Therefore, these results indicate that RS post-transcriptionally controls the mRNA levels of chloroplast proteins.

### 3.5 Light antagonizes regulation of RS-regulated AS events

**Figure 6:**
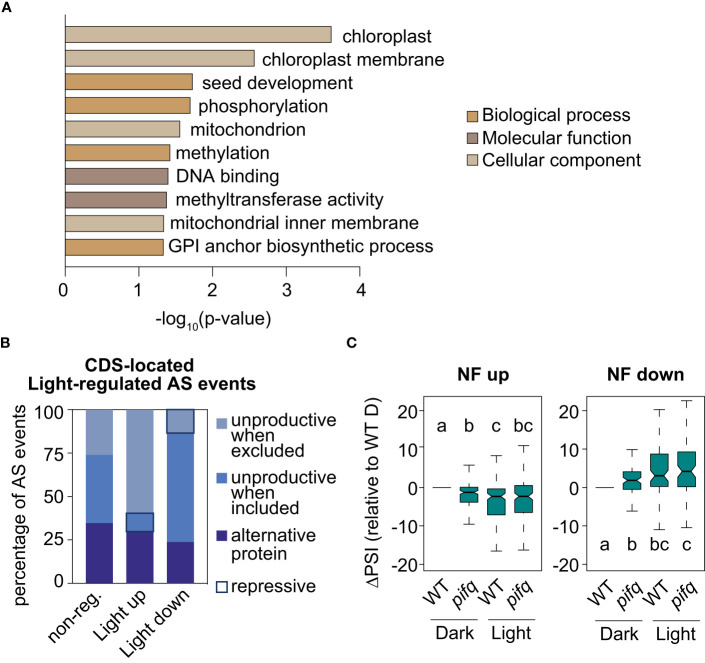
Light regulation of NF-regulated AS events. **(A)** Enriched gene ontology categories of the 247 genes defined as differentially spliced between dark and light conditions. DAVID p-value indicates significance (Fisher’s exact test; *P* < 0.05). **(B)** Percentage of light-regulated AS events located in the coding gene region that potentially produce unproductive mRNAs or alternative protein isoforms (see Material and Methods for details) in three different groups of AS events: non-regulated (non-reg.), up- or downregulated by light. Repressive AS events are highlighted. **(C)** Boxplot representation of the ∆PSI between wild-type (WT) and *pifq* seedlings grown in continuous light or darkness in respect of dark-grown WTs (WT D) of AS events up- or downregulated by norflurazon (NF). Letters denote the statistically significant differences between medians (Kruskal-Wallis; *P* < 0.05).

Retrograde and light signals converge to antagonistically regulate transcription of a subset of common genes (Ruckle et al., 2012; Martín et al., 2016; Xu et al., 2016). Given this preceding information, the molecular link between retrograde and light signaling at the RNA level was investigated. First, by following the same criteria as those established for identifying RS-regulated AS events (see Material and Methods), the splicing profiles of 3-day-old WT seedlings grown either in continuous darkness or white light were compared (GSE164122; Martín and Duque, 2021). This comparison resulted in the definition of 309 light-regulated AS events (**Supplemental Table 5**). Importantly, the GO analysis of genes harboring these AS events also revealed enrichment for chloroplast proteins (**Figure 6A**). Moreover, the study of the predicted impact on the ORFs of the 246 light-regulated AS events located in the CDS unveiled that light enhances inclusion of alternative sequences generating unproductive mRNAs when the sequence is excluded, while it downregulates inclusion of unproductive sequences when it is included (**Figure 6B**). Hence, a minority of AS events produce unproductive mRNAs in the light (repressive AS events; **Figure 6B** and Hartmann et al., 2016), while most do so in the presence of norflurazon (repressive AS events; **Figure 4B**). This result indicates that RS- and light-mediated AS exert opposite molecular functions, respectively enhancing and diminishing the accumulation of unproductive transcripts. Interestingly, some of the NF-regulated AS genes mentioned, such as *U2AF65B*, *GRP8* and *PPL1* have also been characterized as being differentially spliced between dark and light conditions (Hartmann et al., 2016). To assess whether light globally influences NF-regulated AS events, their inclusion levels were quantified in 3-day-old WT and *pifq* seedlings grown in continuous darkness or light (GSE164122; Martín and Duque, 2021). NF-downregulated and NF-upregulated AS events were respectively upregulated or downregulated by light in WT seedlings (**Figure 6C**). Further validating the influence of light in the AS of these events, the inclusion levels of these AS events had the same trend in dark-grown *pifq* seedlings, known to mimic light responses both at the molecular and phenotypic level (Leivar et al., 2008; Leivar et al., 2009). This result firmly revealed that RS-regulated AS events are antagonistically regulated by light, which is in line with the opposite molecular functions of RS- and light-regulated AS events (**Figures 4B**, **6B**), and also, with the common enrichment of chloroplast genes among the sets of RS- and light-regulated AS genes (**Figures 5A**, **6A** and **Supplemental Figure 12**).

## 4 Discussion

Light and chloroplast-to-nucleus retrograde signals converge to control transcription of genes involved in different aspects of plant photomorphogenesis, from morphogenesis to chloroplast biogenesis and functioning. Both signaling pathways coordinate the expression of photomorphogenic genes through the control of common regulators such as the GLK1 transcription factor (Martín et al., 2016). Strikingly, the results presented here indicate that splicing of RS-regulated AS events is also controlled by light (**Figure 6C**), implying that the molecular convergence extends beyond transcriptional regulation (**Supplemental Figure 14**). Noteworthy, as described for transcription, these signaling pathways have antagonistic effects: while RS induces the inclusion of specific AS events, light represses it, and vice versa (**Figure 6C** and **Supplemental Figure 14**). Consequently, this antagonistic regulation implements opposite molecular outcomes: retrograde signals induce accumulation of unproductive transcripts (**Figure 4B**), which are globally targeted to degradation through the NMD surveillance pathway (**Figure 4C**), while light represses this process (**Figure 6B**). Importantly, a paper published in 2014 showed that light-induced AS of particular genes was not dependent on the light perception triggered by photoreceptors, but instead, on functional chloroplasts, demonstrating a role for RS in light-mediated AS (Petrillo et al., 2014). This raises the possibility that the light control of RS-regulated AS events shown in this article (**Figure 6C**) does not occur as a result of the molecular pathways activated by plant photoreceptors, but as a consequence of retrograde signals operating from chloroplasts. This possibility would imply that the two pathways share their molecular targets without connecting their signaling proteins. However, the molecular studies addressing how retrograde and light signals control transcription have extensively demonstrated the existence of proteins that interconnect both signaling pathways (Griffin and Toledo-Ortiz, 2022), thus indicating a connection upstream of their target genes. A deep understanding of the molecular regulators implementing splicing responses to retrograde and light signals is crucial to determine at which step of the post-transcriptional regulation both signaling pathways converge.

Interestingly, the genes that are differentially spliced by retrograde signals are often involved in photomorphogenesis, which is in line with RS and light having respectively a negative and positive role on them. First, there is a great overrepresentation of genes encoding for proteins related to different aspects of the functioning and development of chloroplasts (**Figures 5A**, **6A** and **Supplementary Figures 12**, **13**). Furthermore, key regulators of the light-regulated morphogenic pattern, such as *DET1* (DE-ETIOLATED 1; Chory et al., 1989) and *SHW1* (SHORT HYPOCOTYL IN WHITE LIGHT 1; Bhatia et al., 2008), were identified. Therefore, a precise regulation of splicing on these transcripts might be crucial for seedlings to develop photomorphogenically. In agreement, a small number of functional studies have already demonstrated the participation of individual splicing isoforms in photomorphogenesis (Shikata et al., 2014; Hartmann et al., 2016; Dong et al., 2020).

In addition, this study also revealed a subset of AS genes whose mRNA levels change in response to retrograde signals (**Figure 4D**). This fact can result from overlapping transcriptional and splicing-associated post-transcriptional mechanisms that share their target genes. In this case, AS would reinforce transcriptional mechanisms to, probably, act as a fail-safe mechanism that ensures regulation of the mRNA levels of specific transcripts. By the contrary, changes in the mRNA levels can just reflect the consequences of being differentially spliced but not differentially transcribed. This second scenario would imply that each regulatory mechanism act over a different set of genes, although, as in the case reported here, with similar biological functions: genes encoding for chloroplast proteins (**Figures 5A**, **6A** and **Supplemental Figure 12**).

Overall, this study reveals that changes in AS induced by RS have a negative impact on the expression of chloroplast proteins. Therefore, under environmental or developmental contexts implying dysfunctional chloroplasts, AS will act in conjunction with transcriptional regulation to repress the mRNA levels of genes encoding for chloroplast proteins (**Supplemental Figure 14**). Moreover, this work exemplifies the importance of conducting molecular studies that integrate both transcriptional and post-transcriptional regulatory mechanisms, which are indeed interrelated processes, to obtain a complete and precise comprehension of gene expression regulation.

## Data availability statement

The datasets presented in this study can be found in online repositories. The names of the repository/repositories and accession number(s) can be found in the article/**Supplementary Material**.

## Author contributions

The author confirms sole responsibility for the following: study conception, analysis and interpretation of results, and manuscript preparation.

## Supplementary material

Supplementary Figure 1 Comparison between AS events defined using individual or merged replicates. Proportion of each type of AS event for which inclusion is differentially up- or downregulated by norflurazon (NF; top), lincomycin (Linc; middle) or light (bottom), when comparing individual or merged samples. The total number of AS events in each comparison is indicated. ALT5, alternative 5´splice site; ALT3, alternative 3´splice site; IR, intron retention; ES, exon skipping.

Supplementary Figure 2 mRNA level quantification of photosynthesis-associated nuclear genes. Graph bars showing the expression levels from our mRNA quantification (cRPKM; see Materials and Methods for details) of a subset of representative photosynthesis-associated nuclear genes (*PhANGs*; Allen et al., 2003) in wild-type (WT) and *gun1* samples treated or not with norflurazon (NF).

Supplementary Figure 3 Overlap between genes defined as NF-regulated in distinct analyses. Venn diagram representing the overlap between genes defined as regulated by norflurazon (NF) in this analysis and the one conducted in Xiaobo et al., 2019. For genes defined as NF-regulated in Xiaobo et al., 2019, we only assessed overlap of those that fulfilled the coverage criteria used in our expression analysis (see Materials and Methods for details).

Supplementary Figure 4 Coverage plot of NF-regulated AS events. Read density histograms of RNA-seq data comparing WT and *gun1* seedlings treated or not with norflurazon for an AS event of each type: ALT5, alternative 5´splice site; ALT3, alternative 3´splice site; IR, intron retention; ES, exon skipping. The associated gene models are shown at the bottom of each coverage plot.

Supplementary Figure 5 NF-mediated AS and GE responses in the *gun1* mutant. (A) Boxplot representation of the ΔPSI (top) and log2 fold change (FC; bottom) between norflurazon (NF) and control samples of genes differentially spliced (top) or expressed (bottom) in wild-type (WT) and *gun1* samples.

Supplementary Figure 6 Distribution of the different types of AS events regulated by lincomycin. Number of each type of AS event for which inclusion is differentially up- or downregulated by lincomycin (Linc). Lincomycin non-regulated AS events represent the proportion of the different types of AS event in the genome (see Material and Methods for details). ALT5, alternative 5´splice site; ALT3, alternative 3´splice site; IR, intron retention; ES, exon skipping.

Supplementary Figure 7 Genomic regulatory features associated with differentially spliced introns and exons. Distributions of the length and GC content of the alternatively spliced introns **(A)** or exons **(B)** and the respective surrounding exons or introns, together with the splicing site strength of their 5´ and 3´ splice sites. Non-regulated sets contain introns **(A)** or exons **(B)** fulfilling our read coverage criteria used for AS analysis (see Material and Methods for details) and not being differentially spliced by norflurazon (NF). Asterisks indicate statistically significant differences in respect of the control set (Mann-Whitney U test; *, *P* < 0.05; **, *P* < 0.01; ***, *P* < 0.001; n. s., non-significant).

Supplementary Figure 8 GCC content of differentially spliced exons and introns. Distributions of the ratio between the GCC content of alternative introns and their surrounding exons (top), or between the alternative exons and their surrounding introns (bottom). Non-regulated sets contain the introns (top) or exons (bottom) fulfilling our read coverage criteria used for AS analysis (see Material and Methods for details) and not being differentially spliced. Asterisks indicate statistically significant differences in respect of the control sets (Mann-Whitney U test; *, *P* < 0.05; **, *P* < 0.01; ***, *P* < 0.001; n.s., non-significant).

Supplementary Figure 9 NMD regulation of NF-regulated AS events predicted to generate alternative protein isoforms. Boxplot representation of the percent of inclusion (PSI) values of the NF-regulated AS events predicted to generate alternative proteins in wild-type (WT), *upf1*, *upf3* and *upf1upf3* seedling samples. Statistically significant differences are calculated in respect of the WT (Mann-Whitney U test; n. s., non-significant). NF, norflurazon.

Supplementary Figure 10 Overlap between genes differentially expressed and spliced in response to norflurazon. **(A)** Venn diagram representing the overlap between genes defined as differentially expressed or differentially spliced in response to norflurazon (NF) **(B)** Percentage of genes regulated or not by splicing (non-reg.), whose mRNA levels are up-, downregulated or not regulated in response to norflurazon (see Material and Methods for details in the gene expression analysis).

Supplementary Figure 11 mRNA level quantification of genes harboring repressive AS events. Graph bars showing the expression levels from our mRNA quantification (cRPKM; see Materials and Methods for details) of a subset of genes harboring repressive AS events in wild-type (WT) and *gun1* samples treated or not with norflurazon (NF).

Supplementary Figure 12 Functional analysis of genes differentially spliced in response to lincomycin. Enriched gene ontology categories of the 310 genes defined as differentially spliced in response to lincomycin. DAVID p-value indicates significance (Fisher’s exact test; *P* < 0.05).

Supplementary Figure 13 Subcellular localization of genes differentially spliced in response to retrograde signals. Subcellular localization of genes differentially spliced in response to norflurazon (AS genes), in comparison with the set of genes not regulated by AS (non-regulated genes; see Material and Methods for details). This classification is based on the Araport 11 subcellular predictions available at TAIR (http://www.arabidopsis.org). The percentage of genes encoding for chloroplast-located proteins for each set is indicated. Asterisks indicate statistically significant differences between the two set of genes (two-sided Fisher’s test; **, *P* < 0.01; ***, *P* < 0.001; n.s., non-significant).

Supplementary Figure 14 Proposed model for the regulation of chloroplast protein production by AS in response to retrograde and light signals. Genes encoding chloroplast proteins are antagonistically regulated by the action of transcription factors (TF) controlled by light and retrograde signals (Martín et al., 2016; Xu et al., 2016). This study demonstrates a molecular convergence of these molecular pathways also at the splicing level. Retrograde signals, through the action of GUN1, induce accumulation of splicing variants targeted by the nonsense-mediated decay RNA pathway (NMD). On the contrary, light represses their accumulation.

Supplementary Table 1 List of genes whose mRNA levels are differentially regulated by norflurazon in wild-type seedlings. Columns indicate the gene locus, name and expression values (cRPKMs) in 5-day-old wild-type (WT) and *gun1* seedlings treated with norflurazon (NF) and untreated WTs. See Material and Methods for details on cRPKM values.

Supplementary Table 2 Summary of the sequencing data used in this study. Details of the 24 samples obtained from the NCBI Short Read Archive (SRA) and used in this study. For each sample this information includes i) GEO accession number, ii) sequencing platform, iii) mRNA purification method, iv) name of the individual and merged samples, v) number (ReadN) and length of reads, and vi) percentage of reads mapping unique and multiple times at the genome or reference transcriptome (TAIR10 annotation from Ensembl Plants v31).

Supplementary Table 3 List of AS events differentially regulated by norflurazon in wild-type seedlings. Columns indicate information associated to each AS event including the locus, the *vast-tools* event identifier, the genomic coordinates, the length in pair bases, the type of event and the percent of inclusion (PSI) in 5-day-old wild-type (WT) and *gun1* seedlings treated with norflurazon (NF) and untreated WTs. NA for PSI values indicates insufficient read coverage (see Material and Methods for details).

Supplementary Table 4 List of AS events differentially regulated by lincomycin in wild-type seedlings. Columns indicate information associated to each AS event including the locus, the *vast-tools* event identifier, the genomic coordinates, the length in pair bases, the type of event and the percent of inclusion (PSI) in 5-day-old wild-type (WT) seedlings treated or not with lincomycin (Linc).

Supplementary Table 5 List of AS events differentially regulated by light in dark-grown wild-type seedlings. Columns indicate information associated to each AS event including the locus, the *vast-tools* event identifier, the genomic coordinates, the length in pair bases, the type of event and the percent of inclusion (PSI) in wild-type (WT) seedlings grown in continuous darkness (D) or white light (WL) for 3 days.
