# Supplementary figures and images for: Regulation of alternative splicing by retrograde and light signals converges to control chloroplast proteins
Source: Front Plant Sci. 2023 Feb 10;14:1097127. doi: 10.3389/fpls.2023.1097127 (PMC9950775; doi:10.3389/fpls.2023.1097127)

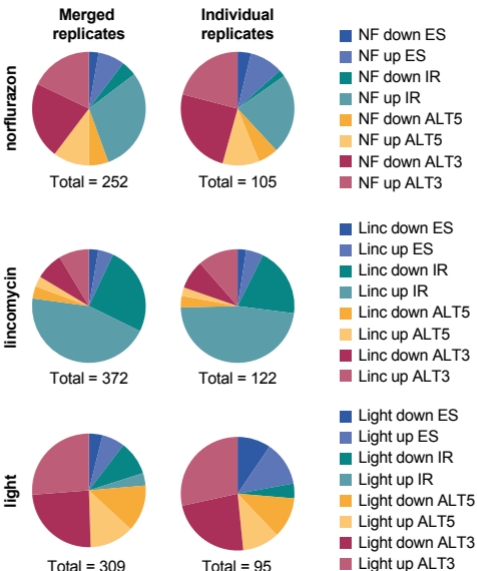

Supplement: Supplementary Figure 1 — Comparison between AS events defined using individual or merged replicates. Proportion of each type of AS event for which inclusion is differentially up- or downregulated by norflurazon (NF; top), lincomycin (Linc; middle) or light (bottom), when comparing individual or merged samples. The total number of AS events in each comparison is indicated. ALT5, alternative 5´splice site; ALT3, alternative 3´splice site; IR, intron retention; ES, exon skipping. [file Image_1.pdf]

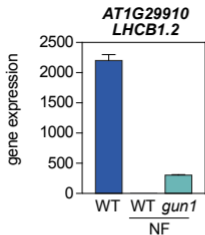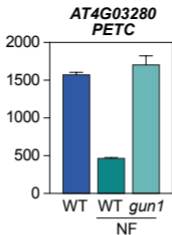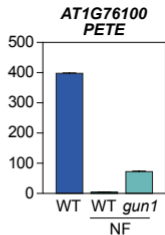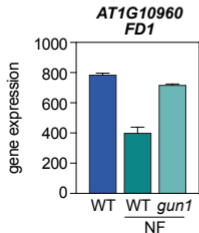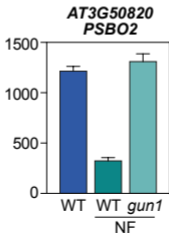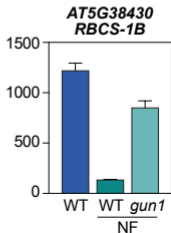

Supplement: Supplementary Figure 2 — mRNA level quantification of photosynthesis-associated nuclear genes. Graph bars showing the expression levels from our mRNA quantification (cRPKM; see Materials and Methods for details) of a subset of representative photosynthesis-associated nuclear genes (PhANGs; Allen et al., 2003) in wild-type (WT) and gun1 samples treated or not with norflurazon (NF). [file Image_2.pdf]

**NF-regulated  
in Xiabo et al. 2019**

**NF-regulated  
in this analysis**

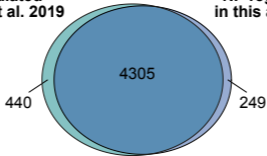

Supplement: Supplementary Figure 3 — Overlap between genes defined as NF-regulated in distinct analyses. Venn diagram representing the overlap between genes defined as regulated by norflurazon (NF) in this analysis and the one conducted in Xiaobo et al., 2019. For genes defined as NF-regulated in Xiaobo et al., 2019, we only assessed overlap of those that fulfilled the coverage criteria used in our expression analysis (see Materials and Methods for details). [file Image_3.pdf]

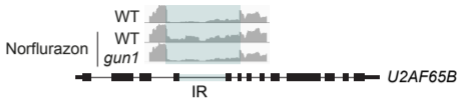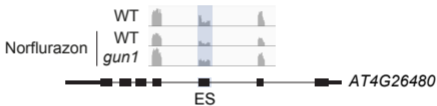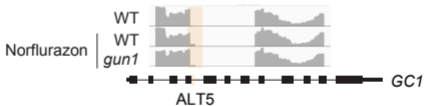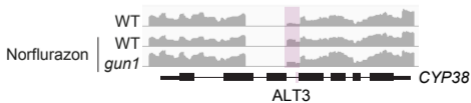

Supplement: Supplementary Figure 4 — Coverage plot of NF-regulated AS events. Read density histograms of RNA-seq data comparing WT and gun1 seedlings treated or not with norflurazon for an AS event of each type: ALT5, alternative 5´splice site; ALT3, alternative 3´splice site; IR, intron retention; ES, exon skipping. The associated gene models are shown at the bottom of each coverage plot. [file Image_4.pdf]

**A**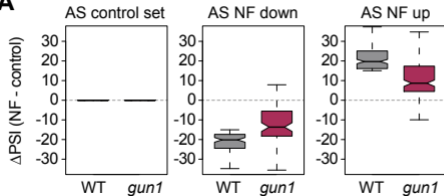**B**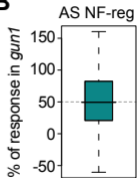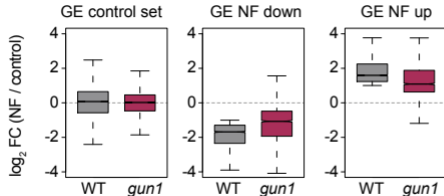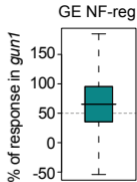

Supplement: Supplementary Figure 5 — NF-mediated AS and GE responses in the gun1 mutant. (A) Boxplot representation of the ΔPSI (top) and log2 fold change (FC; bottom) between norflurazon (NF) and control samples of genes differentially spliced (top) or expressed (bottom) in wild-type (WT) and gun1 samples. [file Image_5.pdf]

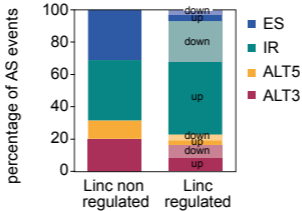

Supplement: Supplementary Figure 6 — Distribution of the different types of AS events regulated by lincomycin. Number of each type of AS event for which inclusion is differentially up- or downregulated by lincomycin (Linc). Lincomycin non-regulated AS events represent the proportion of the different types of AS event in the genome (see Material and Methods for details). ALT5, alternative 5´splice site; ALT3, alternative 3´splice site; IR, intron retention; ES, exon skipping. [file Image_6.pdf]

**A**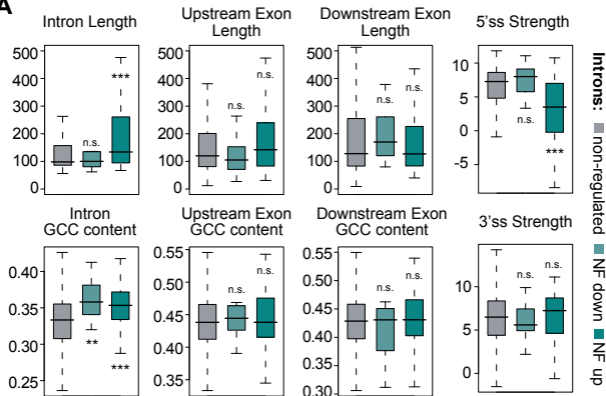**B**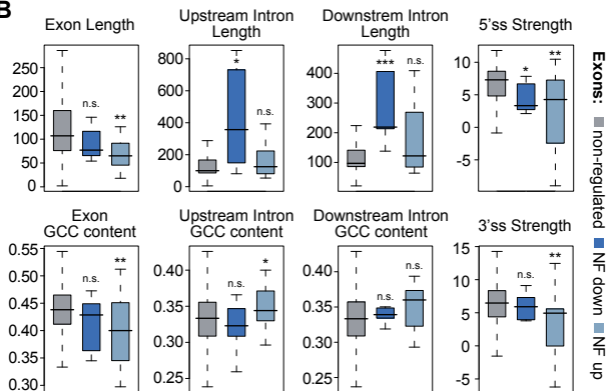

Supplement: Supplementary Figure 7 — Genomic regulatory features associated with differentially spliced introns and exons. Distributions of the length and GC content of the alternatively spliced introns (A) or exons (B) and the respective surrounding exons or introns, together with the splicing site strength of their 5´ and 3´ splice sites. Non-regulated sets contain introns (A) or exons (B) fulfilling our read coverage criteria used for AS analysis (see Material and Methods for details) and not being differentially spliced by norflurazon (NF). Asterisks indicate statistically significant differences in respect of the control set (Mann-Whitney U test; *, P < 0.05; **, P < 0.01; ***, P < 0.001; n. s., non-significant). [file Image_7.pdf]

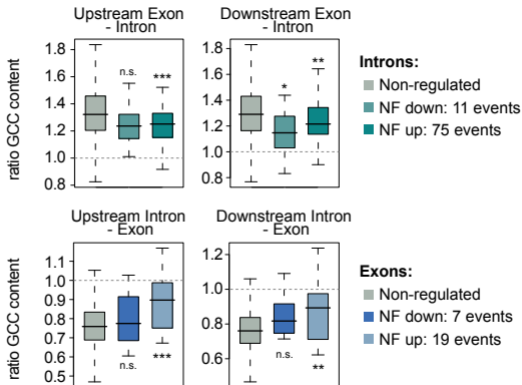

Supplement: Supplementary Figure 8 — GCC content of differentially spliced exons and introns. Distributions of the ratio between the GCC content of alternative introns and their surrounding exons (top), or between the alternative exons and their surrounding introns (bottom). Non-regulated sets contain the introns (top) or exons (bottom) fulfilling our read coverage criteria used for AS analysis (see Material and Methods for details) and not being differentially spliced. Asterisks indicate statistically significant differences in respect of the control sets (Mann-Whitney U test; *, P < 0.05; **, P < 0.01; ***, P < 0.001; n.s., non-significant). [file Image_8.pdf]

**NF-regulated  
alternative protein  
AS events**

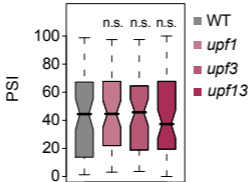

Supplement: Supplementary Figure 9 — NMD regulation of NF-regulated AS events predicted to generate alternative protein isoforms. Boxplot representation of the percent of inclusion (PSI) values of the NF-regulated AS events predicted to generate alternative proteins in wild-type (WT), upf1, upf3 and upf1upf3 seedling samples. Statistically significant differences are calculated in respect of the WT (Mann-Whitney U test; n. s., non-significant). NF, norflurazon. [file Image_9.pdf]

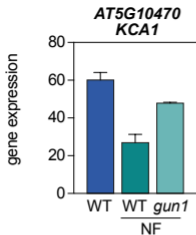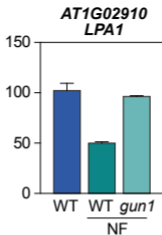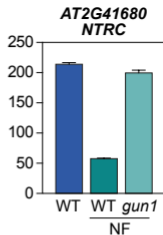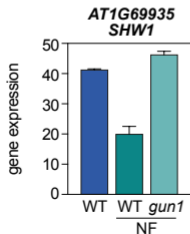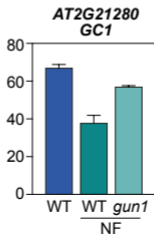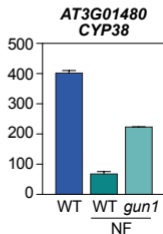

Supplement: Supplementary Figure 11 — mRNA level quantification of genes harboring repressive AS events. Graph bars showing the expression levels from our mRNA quantification (cRPKM; see Materials and Methods for details) of a subset of genes harboring repressive AS events in wild-type (WT) and gun1 samples treated or not with norflurazon (NF). [file Image_11.pdf]

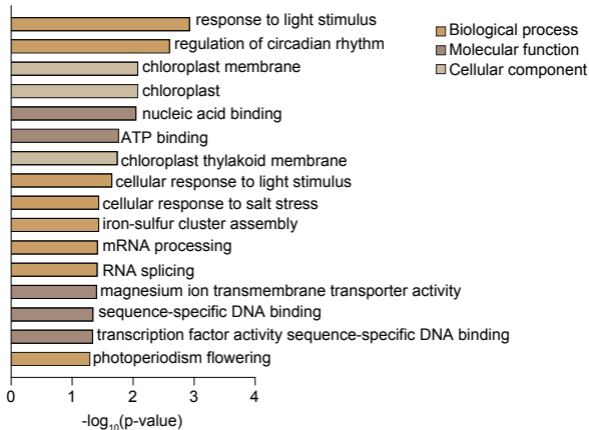

Supplement: Supplementary Figure 12 — Functional analysis of genes differentially spliced in response to lincomycin. Enriched gene ontology categories of the 310 genes defined as differentially spliced in response to lincomycin. DAVID p-value indicates significance (Fisher’s exact test; P < 0.05). [file Image_12.pdf]

**Retrograde Signals**

**Light Signals**

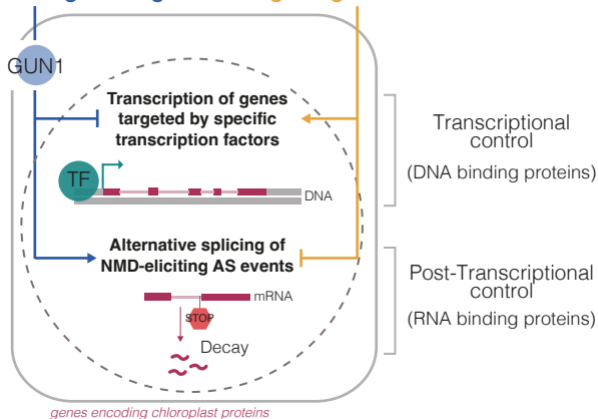

Supplement: Supplementary Figure 14 — Proposed model for the regulation of chloroplast protein production by AS in response to retrograde and light signals. Genes encoding chloroplast proteins are antagonistically regulated by the action of transcription factors (TF) controlled by light and retrograde signals (Martín et al., 2016; Xu et al., 2016). This study demonstrates a molecular convergence of these molecular pathways also at the splicing level. Retrograde signals, through the action of GUN1, induce accumulation of splicing variants targeted by the nonsense-mediated decay RNA pathway (NMD). On the contrary, light represses their accumulation. [file Image_14.pdf]
