# Supplementary material for: Regulation of alternative splicing by retrograde and light signals converges to control chloroplast proteins
Source: Front Plant Sci. 2023 Feb 10;14:1097127. doi: 10.3389/fpls.2023.1097127 (PMC9950775; doi:10.3389/fpls.2023.1097127)
Supplement: Supplementary Figure 10 — Overlap between genes differentially expressed and spliced in response to norflurazon. (A) Venn diagram representing the overlap between genes defined as differentially expressed or differentially spliced in response to norflurazon (NF) (B) Percentage of genes regulated or not by splicing (non-reg.), whose mRNA levels are up-, downregulated or not regulated in response to norflurazon (see Material and Methods for details in the gene expression analysis). [file Image_10.pdf]

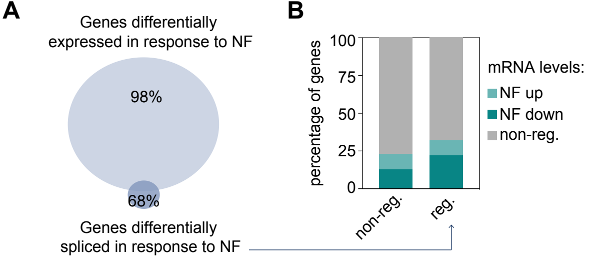
A
Genes differentially expressed in response to NF
98%
68%
Genes differentially spliced in response to NF
B
percentage of genes
100
75
50
25
0
non-reg.
reg.
mRNA levels:
NF up
NF down
non-reg.
