# Supplementary material for: Regulation of alternative splicing by retrograde and light signals converges to control chloroplast proteins
Source: Front Plant Sci. 2023 Feb 10;14:1097127. doi: 10.3389/fpls.2023.1097127 (PMC9950775; doi:10.3389/fpls.2023.1097127)
Supplement: Supplementary Figure 13 — Subcellular localization of genes differentially spliced in response to retrograde signals. Subcellular localization of genes differentially spliced in response to norflurazon (AS genes), in comparison with the set of genes not regulated by AS (non-regulated genes; see Material and Methods for details). This classification is based on the Araport 11 subcellular predictions available at TAIR (http://www.arabidopsis.org). The percentage of genes encoding for chloroplast-located proteins for each set is indicated. Asterisks indicate statistically significant differences between the two set of genes (two-sided Fisher’s test; **, P < 0.01; ***, P < 0.001; n.s., non-significant). [file Image_13.pdf]

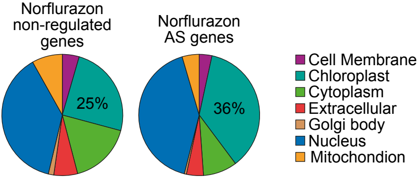
Norflurazon non-regulated genes
25%
Norflurazon AS genes
36%
Cell Membrane
Chloroplast
Cytoplasm
Extracellular
Golgi body
Nucleus
Mitochondion
